# From the Matrix to the Nucleus and Back: Mechanobiology in the Light of Health, Pathologies, and Regeneration of Oral Periodontal Tissues

**DOI:** 10.3390/biom11060824

**Published:** 2021-05-31

**Authors:** Martin Philipp Dieterle, Ayman Husari, Thorsten Steinberg, Xiaoling Wang, Imke Ramminger, Pascal Tomakidi

**Affiliations:** 1 Center for Dental Medicine, Division of Oral Biotechnology, Medical Center—University of Freiburg, Faculty of Medicine, University of Freiburg, Hugstetterstr. 55, 79106 Freiburg, Germany; martin.dieterle@uniklinik-freiburg.de (M.P.D.); xiaoling.wang@uniklinik-freiburg.de (X.W.); imke.ramminger@uniklinik-freiburg.de (I.R.); pascal.tomakidi@uniklinik-freiburg.de (P.T.); 2 Center for Dental Medicine, Department of Orthodontics, Medical Center—University of Freiburg, Faculty of Medicine, University of Freiburg, Hugstetterstr. 55, 79106 Freiburg, Germany; ayman.husari@uniklinik-freiburg.de; 3 Faculty of Engineering, University of Freiburg, Georges-Köhler-Allee 101, 79110 Freiburg, Germany

**Keywords:** mechanotransduction (MT), nuclear mechanotransduction (NMT), YAP/TAZ, extracellular matrix (ECM), gingipain proteases, periodontitis, oral squamous cell carcinoma (OSCC), regeneration

## Abstract

Among oral tissues, the periodontium is permanently subjected to mechanical forces resulting from chewing, mastication, or orthodontic appliances. Molecularly, these movements induce a series of subsequent signaling processes, which are embedded in the biological concept of cellular mechanotransduction (MT). Cell and tissue structures, ranging from the extracellular matrix (ECM) to the plasma membrane, the cytosol and the nucleus, are involved in MT. Dysregulation of the diverse, fine-tuned interaction of molecular players responsible for transmitting biophysical environmental information into the cell’s inner milieu can lead to and promote serious diseases, such as periodontitis or oral squamous cell carcinoma (OSCC). Therefore, periodontal integrity and regeneration is highly dependent on the proper integration and regulation of mechanobiological signals in the context of cell behavior. Recent experimental findings have increased the understanding of classical cellular mechanosensing mechanisms by both integrating exogenic factors such as bacterial gingipain proteases and newly discovered cell-inherent functions of mechanoresponsive co-transcriptional regulators such as the Yes-associated protein 1 (YAP1) or the nuclear cytoskeleton. Regarding periodontal MT research, this review offers insights into the current trends and open aspects. Concerning oral regenerative medicine or weakening of periodontal tissue diseases, perspectives on future applications of mechanobiological principles are discussed.

## 1. Introduction

Within tissues, cells are embedded in an extracellular matrix (ECM), consisting of both fibrous proteins similar to collagen and the ground substance, which is mainly made up of proteoglycans and glycoproteins [1]. Tissue homeostasis and regeneration is governed by a plethora of different signals, involving differentiation and growth factors, endocrine signals, and neuronal stimuli, which have been extensively studied in vitro and in vivo [2,3,4,5,6]. Above, the cells are in close contact with neighboring cells for the purpose of metabolic coupling and, in the case of epithelia, to enclose a defined physiological milieu [7,8]. Thus, a complex, three-dimensional environment is not only responsible for histogenesis during development, but also for tissue homeostasis and regeneration [9,10].

This intimate relationship between cells and their ECM makes it necessary that signals are exchanged between both compartments. Beneath biochemical factors and ion currents, mechanical cues are transmitted from the ECM to the cell and vice versa within a process called mechanotransduction (MT). The same principles apply to cell-to-cell contact sites, where cellular traction forces are transmitted between adjacent cells [11,12,13,14,15].

On the molecular level, MT relies on membrane-embedded cellular receptor proteins that sense and bind to extracellular ligands, which are either ECM proteins in the case of cell-to-matrix adhesion or cell-bound ligands in the case of cell-to-cell contacts [11,12,13,14,15,16].

Focal adhesions (FAs) are microscopically detectable contact sites between cells and the ECM and consist of membrane-bound receptor proteins, called integrins, which can bind to collagens, fibronectins, and various other ECM components [15]. Movements of the ECM, such as shear stress or compression forces, induce the formation and consecutive strengthening of FAs, which corresponds with mechanobiological cellular adaptation processes [17]. Intracellularly, integrins are connected to diverse adaptor proteins, which constitute a versatile signaling scaffold to address many different signaling pathways in response to mechanical loading [15]. Finally, these cascades converge on actin-regulating proteins (ARPs) as well as mechanosensitive co-transcriptional activators, such as the Yes-associated protein 1 (YAP1, henceforth designated as YAP) [18,19]. Conversely, actin- and myosin-dependent cellular traction forces are fed back to the ECM via integrins, rendering FAs an outside-in and inside-out signaling hub [20,21].

Cell-to-cell contacts are mostly established by homophilic and Ca^2+^-dependent cellular adhesion proteins, namely members of the cadherin (Cad) families. The histological equivalents of these connections are called adherens junctions (AJs) and are indispensable for epithelial integrity and barrier functions. Similar to FAs, AJs are intracellularly coupled to further signal-transducing proteins comprising members of the catenin family and the actin cytoskeleton [22,23,24].

Aside from the cytoplasmic cytoskeleton, more and more experimental evidence proves the enormous contribution of nuclear mechanics and the nuclear cytoskeleton to cellular MT, genomic integrity, and tissue regeneration [25,26]. In particular, nuclear lamins, and their role in epigenetic regulation and chromatin structure, are tightly interrelated to mechanobiological processes [27,28]. Thus, the concept of nuclear mechanotransduction (NMT) widens the above-described ECM-cytosolic network to an integrative ECM-cytosol-nucleus network, involved in cellular key functions as diverse as proliferation, migration, differentiation, and apoptosis. Therefore, understanding the key concepts which govern cellular MT is undoubtedly a prerequisite for a deeper understanding of periodontal tissue homeostasis, regeneration, and disease.

Within the oral cavity, the periodontal structures consisting of the gingiva, the periodontal ligament (PDL), cementum and the alveolar bone are permanently subjected to cyclic and static mechanical loading, which is a direct consequence of chewing, occlusion, mastication or orthodontic treatment [29]. Although the ECMs of the periodontium harbor different biochemical and biophysical properties, their mechanical integrity and continuous remodeling and regeneration is a basic requirement for oral health [30,31,32]. Imbalances in matrix metalloproteinase (MMP) and the tissue-inhibitor of metalloproteinases (TIMPs) regulation or the presence of pathogen-derived proteases such as the *Porphyromonas gingivalis* (*P. gingivalis*) gingipains can severely damage the periodontium and lead to inflammatory processes with consecutive catabolic degradation of the ECM [33,34,35,36]. This, of course, changes the mechanosignaling pathways and thus influences cell behavior. In conjunction with the host’s inflammatory response, their susceptibility to pathogen-induced damage, and further microbe-derived virulence factors can contribute to or result in the onset or the progression of diseases such as periodontitis or, at worst, even oral squamous cell carcinoma (OSCC) [37,38].

Regarding the various functions of MT in tissue homeostasis, regeneration and health, this review summarizes the molecular principles of core mechanosignaling pathways in the periodontium, including FAs, AJs, YAP1 and NMT. Moreover, potential applications of MT-related processes for periodontal regeneration and disease weakening are described. 

## 2. The ECM, Focal Adhesions and Adherens Junctions in Periodontal Health and Disease

The periodontium is a complex composite tissue consisting of the gingiva, the periodontal ligament (PDL), cementum, and the alveolar bone. Beneath harboring various cell types, amongst others, gingival keratinocytes (GKs), gingival fibroblasts (GFs), periodontal ligament fibroblasts (PDLFs), cementoblasts and osteoblasts, the ECMs of the different periodontal cell and tissue entities are considerably different [39].

The cementum and the alveolar bone consist of an inorganic hydroxyapatite matrix and collagen type I as the major organic compound [40]. Additionally, the glycosaminoglycans (GAGs) decorin and biglycan as well as the glycoproteins (GPs) osteonectin, osteopontin, fibronectin, and osteocalcin can be found in the ECM of both tissues [41,42]. The PDL harbors the fibrous collagen types I and III, and small amounts of collagen V and VI [43]. Above, the basement membrane collagen types IV and VII and collagen type XII, which is important for fibrillar organization, are expressed in the PDL [44]. Elastin, fibronectin (FN) and chondroitin-/dermatan-/keratin-sulfate, containing GAGs, also support PDL function [45].

The gingiva contributes to periodontal integrity via epithelium and connective tissue. While the GKs form a stratified squamous epithelial layer, GFs are embedded in a lamina propria with collagen type I, III, elastin and many other macromolecules [46].

Physical ECM properties are key determinants of cell behavior in vitro and in vivo. The stiffness of the ECM, quantified by the Young’s modulus, as well as viscoelastic properties, spatial arrangement of adhesion points, and other geometric constraints, influence cellular responses through MT and other signaling hubs [14,47,48]. This means that ECM composition, homeostasis and MT are tightly coupled and are, therefore, highly interdependent [49]. Cell morphology, migration, proliferation, differentiation, and apoptosis are consequently not only influenced by biochemical signals, but also by the direct mechanical properties of the respective ECM environment [50]. Interestingly, cellular responses, namely the actomyosin-derived cell-inherent contraction forces, as discussed below, seem to directly reflect ECM stiffness, meaning that the Young’s modulus of the ECM is encoded within the cell’s response to that specific microenvironment [51]. 

Mechanistically, the above-mentioned ECM constituents directly or indirectly, i.e., mediated through adaptor proteins, interact with neighboring cells via surface receptors. Regarding MT, the family of integrin proteins is especially important as they are the core linking hub between the ECM and the cytosol. Within the plasma membrane, integrins form heterodimers, which are composed of an α- and a β-subunit. Various combinations of heterodimers have been described in different experimental systems and they proved to have different ligand specificity. Table 1 summarizes the most important integrin heterodimers and their corresponding ligands relevant to periodontal MT [52,53,54,55,56,57,58]. As can be seen, one heterodimer can sometimes bind more than one ligand (e.g., α1β1-integrin) and one ligand, such as fibronectin, is recognized by multiple heterodimers. Therefore, the tissue- or cell-specific expression pattern of integrins determines its interaction with the ECM. Periodontal cell populations foremostly harbor α2β1, α3β1 and α5β1 integrins. Of interest, the expression pattern of periodontal integrins changes in response to damage or during wound healing or carcinogenesis [59].

Upon mechanical loading, such as during mastication, occlusion or orthodontic treatment, the ECM of the periodontal tissues is deformed. Exemplarily, occlusion forces that exert compressive load onto a tooth are transmitted to the PDL, which serves as a push-pull transducer [60]. This means that the ECM components within the PDL, such as the collagen fibers, are stretched. As they are either directly or indirectly connected to the integrin receptors and these proteins undergo a conformational change, which is herewith the consequence of the initial mechanical stimulus. Therefore, these surface receptors are considered mechanoreceptors, as they transmit the ECM’s physical state into the cell’s interior.

Intracellularly, integrins are linked to various signaling proteins, which function as a molecular clutch that couples integrin–ECM interaction to intracellular biochemical signaling, the centerpiece of MT. Histologically, these integrin-dependent cell-to-matrix connection structures are called FAs [15].

**Figure 1:**
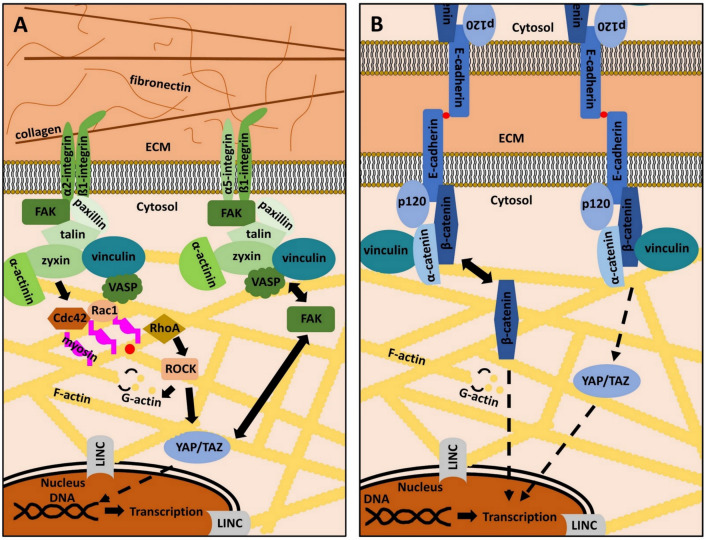
The role of focal adhesions and adherens junctions in mechanotransduction. (**A**): Focal adhesions (FAs) are adhesion structures that bind extracellular matrix (ECM) ligands via integrin receptors. The latter are composed of varying combinations of an α- and a β-subunit. Each heterodimer has specific ECM ligands (see Table 1). Intracellularly, integrins are linked to various signaling molecules that constitute a molecular clutch, which transmits mechanical information from the ECM into the cell’s interior and vice versa. Focal adhesion kinase (FAK), paxillin, talin, zyxin, vinculin, vasodilator-simulated phosphoprotein (VASP) and α-actinin are examples of important FAs proteins, connecting integrin receptors to the actin cytoskeleton (yellow). The small GTP-binding proteins Ras-related C3 botulinum toxin substrate 1 (Rac1), cell division control protein homologue 42 (Cdc42), and Ras homologue A (RhoA), together with Rho-associated, coiled-coil-containing protein kinase (ROCK) modulate the dynamic de- and repolymerization of globular (G)-actin (yellow dots) and filamentous (F)-actin. FAK activity and subcellular localization of yes-associated protein (YAP) and its cellular homologue transcriptional co-activator with PDZ motif (TAZ) are strongly interconnected. The linker of nucleoskeleton and cytoskeleton (LINC) complex couples the cytoplasmic cytoskeleton to the nucleus. Both mechanisms are important to regulate gene expression in response to mechanical signals. Details are described in the main text. (**B**): Cell-to-cell adhesion depends on adherens junctions (AJs). Cadherins, as exemplified by E-Cadherin, are transmembrane proteins that bind other cadherins on neighboring cells in a Ca^2+^-dependent manner (red dots). Intracellularly, cadherins are linked to various proteins, such as p120, α-catenin, β-catenin, and vinculin, which indirectly connect cadherins to the actin cytoskeleton (yellow). YAP/TAZ regulation is also dependent on AJs integrity. β-catenin can also serve as a transcription factor in the nucleus and its subcellular localization contributes to determining cell behavior. Further details are described in the main text.

Next to the plasma membrane, a plethora of proteins form the “integrin signaling layer”, where focal adhesion kinase (FAK), the head domain of talin and paxillin interact with the cytoplasmic integrin domain. Vinculin and the tail of talin are designated as the “force transduction layer” [61]. Finally, these proteins are linked to the actin cytoskeleton through an “actin binding layer”, which consists of α-actinin, vasodilator-simulated phosphoprotein (VASP), and zyxin [62]. Beneath the listed proteins, many other cellular key players, such as sarcoma (Src)-family kinases or the extracellular signal-regulated kinases 1/2 (ERK1/2) have been shown to directly interact with FAs and regulate the activity and assembly status of its components (Figure 1A) [15,63,64].

The actin cytoskeleton is the common downstream target of all MT pathways [65,66]. It not only consists of filamentous (F) actin, but also of actin-regulating proteins (ARPs), which regulate the dynamic building and destruction of the filaments from globular (G) actin monomers. G-actin binds adenosine-triphosphate (ATP) and hydrolyses ATP to adenosine-diphosphate (ADP) within F-actin. This reaction goes along with conformational changes of actin monomers and contributes to the dynamic turnover of actin-related cytoskeletal structures [67,68,69]. Actin polymerization is regulated by a class of guanosine–triphosphate (GTP) binding proteins, known as Ras homologue A (RhoA), cell division control protein homolog 42 (Cdc42), Ras-related C3 botulinum toxin substrate 1 (Rac1), as well as the Rho-associated, coiled-coil-containing protein kinase 1 (ROCK1) (Figure 1A) [70,71,72,73]. These small GTPases are addressed by the mechanotransducing proteins of FAs and their activity state determines the polymerization of G-actin as well as the formation of lamellipodia or filopodia. ROCK1 is even more directly involved in periodontal differentiation, homeostasis, and regeneration, as the inhibition of ROCK1 prevents proper the differentiation of PDL cells into osteoblasts and reduces ECM regeneration via the downregulation of collagen I and fibronectin [74,75]. These findings underscore the essential role of actin cytoskeleton regulation in the MT of the periodontium.

Actin filaments are additionally stabilized by ARPs, such as Arp2/3, tropomyosin or profilin. Contrarily, severing proteins, such as gelsolin, support F-actin depolymerization or destruction [76,77]. Altogether, the complex interplay of actin regulatory proteins governs actin’s dynamic de- and repolymerization and enables complex cellular processes, such as cell division or migration [78]. Further details of these complex cytoskeletal regulatory principles are beyond the scope of this review and interested readers are referred to other comprehensive discussions on this subject [79,80].

Thus far, the description of the actin cytoskeleton and its dynamics does not explain how cells maintain their shape during mechanical stimulation and how cellular integrity can be achieved by mechanisms of FAs-related signaling. To this end, cells need the ability to actively generate forces to withstand external deformation or to exert mechanical stimuli on their environment. This is possible through the action of cytoplasmic motor proteins, known as myosins, which are coupled to the actin cytoskeleton. Via the hydrolysis of ATP, these motor proteins can actively move along actin filaments [81,82,83]. Besides functions in cargo transport, myosins can, therefore, generate tension and traction forces through the relative displacement of actin filaments [84,85,86,87]. This mechanism immediately explains that FAs are not only outside-in signaling platforms that transmit mechanical ECM signals into the cell, but that actomyosin-generated cytoskeletal forces can also be transmitted to the ECM with the help of integrins and their neighboring adaptor proteins. Therefore, FAs are bidirectional mechanosensitive signaling hubs.

Of interest, this is of enormous importance for ECM homeostasis and regeneration in the PDL, as FN and collagen fibrillogenesis depends on intracellularly derived contractile forces. Actin stress fibers serve as a guide trail for the centripetal movement of α5β1-bound FN, which supports FN-FN interactions [88,89]. Collagen molecules can then be deposited on the pre-existing FN fibrils. As this process is force-dependent, characteristic ECM structures, such as the parallel arrays of collagen fibers within the PDL, can be explained via this mechanism and are, therefore, a result of bidirectional MT related to FAs. Interestingly, a recent study gave new insights into the actual nonuniformity of the PDL and revealed the mechanical properties of different subregions within the gomphosis. The so-called collar region is characterized by a high proportion of collagen type I, making it resistant to tensile forces due to high mechanical stiffness. Contrary to that, the furcation region is less stiff and contains less type I collagen, which seems to be associated with a dual function in resisting compressive loads [30].

In periodontitis, collagen and other ECM components are degraded by proteases, such as MMPs or bacteria-derived gingipains (see Section 4) [90]. This changes integrin-dependent MT, leading to a decrease in intracellular actomyosin contractility (outside-in-signaling). Consequently, inside-out signaling is also impaired, which leads to incorrect collagen fibril deposition, worsening the catabolic destruction of the periodontium [36,59].

FAs related signaling also comprise an important step in (alveolar) osteocyte differentiation and the cell’s response to fluid shear stress. The reduction in the protein sclerostin by mechanical loading is mediated by FAK-dependent phosphorylation of the histone deacetylase 5 (HDAC5), which is translocated into the nucleus in response to this post-translational modification. Sclerostin suppression leads to an increase in bone formation and thus mediates an adaptation process by which shear stress leads to mechanical strengthening of the exposed tissue [91,92].

Besides cell-to-matrix adhesion, cell-to-cell adhesion is of great importance, especially in epithelial tissues, such as parts of the gingiva. The main constituents of the so-called adherens junctions (AJs) are cadherin (Cad)-family members (Cads), which are Ca^2+^-dependent, membrane-embedded proteins that connect cells via homophilic interaction [93]. In the cytoplasm, Cads are connected to various proteins, amongst others α-catenin, p120, vinculin or β-catenin [22,94,95,96]. These adaptor proteins are comparable to the integrin-linked intracellular mechanotransducers, as they connect Cads to the actin cytoskeleton. The same mechanisms and principles as discussed above also apply for AJs and qualify the actin cytoskeleton not only as the common final pathway of AJs and FAs signaling, but also as a crosstalk platform that integrates mechanical cues transmitted through various MT pathways [97,98]. ERK1/2, YAP, and its cellular homologue transcriptional co-activator with PDZ motif (TAZ), vinculin and FAK, are both addressed by AJs and FAs, underscoring the complex mutuality of cell-to-cell and cell-to-matrix adhesion (Figure 1B) [17,99,100,101]. This is the reason why current systemic approaches try to elucidate the tissue- and cell-type specific interplay and fine-regulation of signaling crosstalk related to MT [13]. It is of great interest, to shed light into these principles in periodontal tissues.

In the periodontal context, Cads fulfill different functions, ranging from maintenance of cellular differentiation to epithelial barrier function, tumor suppression, and MT-related tissue homeostasis. Specifically, β-catenin, in its function as a transcriptional regulator, is important in PDLF differentiation and simultaneously inhibits the cementoblastic phenotype [102].

The epithelial E-Cad, which is expressed in GKs, plays a significant role both in periodontitis and oral carcinogenesis. Patients suffering from periodontitis show decreased protein levels of E-Cad, which is indirect evidence for a dysfunctional epithelial barrier function. This further promotes the inflammatory process [103]. Downregulation of E-Cad has also been reported in many carcinomas, where it represents a key step during epithelial-to-mesenchymal transition (EMT). Thereby, epithelial cells detach from their surrounding cells and develop a migratory, fibroblastoid phenotype, which is a prerequisite for tissue invasion and metastasis. Regarding oral carcinogenesis, reduced expression levels of E-Cad and β-catenin are indicators of the progression from dysplasia to cancer and an aggressive OSCC phenotype [104]. As chronic inflammation is a risk factor for cancer development, the loss of E-Cad-related barrier function and MT offers valuable insights into the link between periodontitis and OSCC [105,106].

Beneath E-Cad, Cadherin 11 (Cad11) is another member of the cadherin family expressed in periodontal tissues [107,108]. Upon mechanical loading, expression of Cad11 and β-catenin decreases in PDLFs, which consequently leads to a reduction in collagen 1 synthesis and changes in cellular morphology [109]. These findings clearly show that not only FAs but also AJs are involved in ECM homeostasis and regeneration and that cell-to-cell adhesion is not limited to intercellular information exchange. This hypothesis is supported by other experimental results, where knock-down of Cad11 impairs elastin and collagen synthesis [110]. In mice, Cad11 deficiency reduces cell contractility, which again represents the involvement of AJs in both ECM structure and MT [111].

Taken together, the findings presented in this chapter show that the ECM and its molecular composition are important determinants of periodontal cell behavior in the context of MT. As the periodontal tissues are affected by diseases, such as periodontitis or OSCC development, the relevance of MT in these pathophysiological processes is of clinical relevance. So far, the convergence of FAs and AJs-mediated signal transduction on the cytoskeleton has been discussed. However, the mechanisms by which the information encoded within the contractility and tension of the actin-cytoskeleton is translated into cellular adaptation, which depend on further cellular key players, such as the mechanoresponsive co-transcriptional activators YAP/TAZ and the nuclear cytoskeleton, will be discussed in the subsequent sections.

## 3. Mechanotransduction to the Core: YAP/TAZ in the Periodontium

The Yes-associated protein YAP and its cellular homologue TAZ were originally described as transcriptional co-activators in the context of the so-called Hippo signaling pathway. The latter was identified in the fruit fly *Drosophila melanogaster* by mutagenesis screening and the recognition that loss-of-function mutations of certain genes lead to an increase in organ size (overgrowth phenotype) [112]. The core Hippo pathway is evolutionary conserved and its homologues in mammals are involved in many cellular functions, such as proliferation and differentiation, as well as carcinogenesis. The main components are the serine-threonine kinases Ste-20-like kinases 1/2 (Mst1/2) and large tumor suppressor kinase 1/2 (Lats1/2) as well as the above-mentioned co-transcriptional activators, YAP and TAZ [13,113,114,115,116].

Unlike many other phosphorylation cascades, the activity of the Hippo kinases (Hippo signaling turned “on”) leads to the inactivation of the effector proteins YAP/TAZ. This is achieved by the phosphorylation of YAP on Serine127 or TAZ on Serine89, which promotes the association of the proteins with their cytosolic sequestration protein 14-3-3σ [117]. Conversely, the inactivity of the Hippo kinases (Hippo signaling turned “off”) results in a shift of the phosphorylation-dephosphorylation equilibrium towards dephosphorylation, which enables YAP/TAZ to shuttle into the nucleus, where the proteins can exert their function as co-transcriptional activators. Subsequently, depending on the cellular context, YAP/TAZ promote proliferation, differentiation, or many other core physiological functions [114,117]. The slightly non-intuitive mechanism is easily understood by having a look at regulatory upstream signals that govern the activity of the Mst1/2 and Lats1/2 kinases. For example, in the apical junctional region of the epithelia, proteins such as Merlin/neurofibromatosis 2 (NF2), KIBRA, and Salvador-homologue 1 (Sav1) form a protein complex that responds to cell-to-cell adhesion. Therefore, MT-relevant signals, such as a high cell density, activate the Mst1/2 and Lats1/2 kinases via this junctional complex, and thereby inhibit YAP/TAZ translocation into the nucleus, which in turn inhibits proliferation and organ overgrowth [118,119].

Apart from canonical Hippo signaling, YAP/TAZ are also regulated by various other upstream signals, which are at least in part independent of the above-discussed kinase mechanisms. Signals from G-protein coupled receptors (GPCRs) as well as receptor tyrosine kinases (RTKs) and receptor serine/threonine kinases also converge on YAP/TAZ, as exemplified by their regulation through platelet-derived growth factor (PDGF) or transforming growth factor beta (TGF-β) [120,121,122].

**Figure 2:**
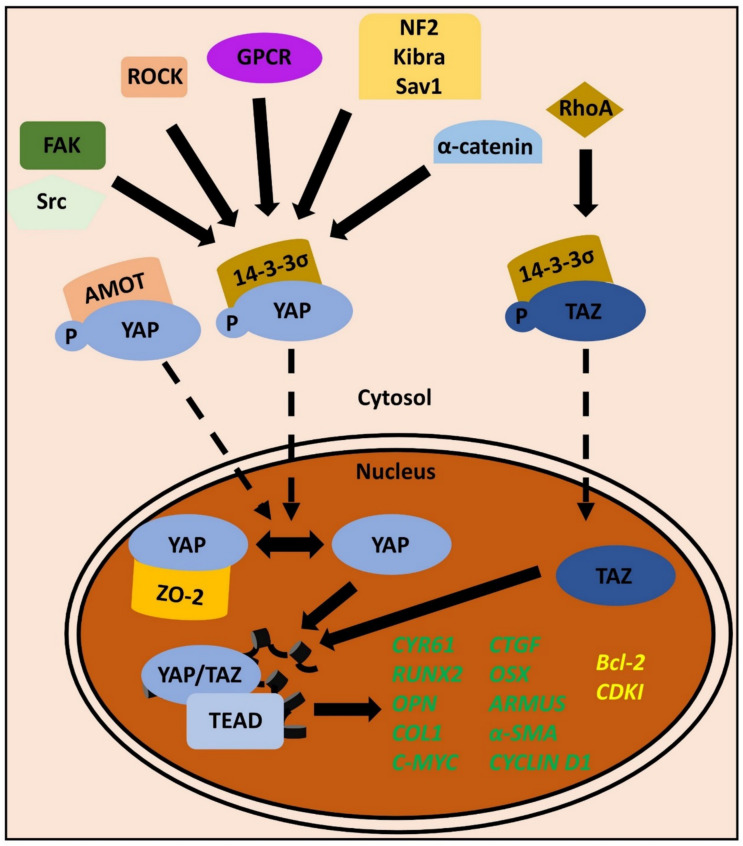
The transcriptional co-activators YAP and TAZ are regulated by many cellular key players. Yes-associated protein (YAP) and its cellular homologue transcriptional co-activator with PDZ motif (TAZ) are master-regulators of cellular mechanotransduction. A plethora of upstream signals converge on these proteins. Nonetheless, there seems to be slight differences in the exact cellular functions of YAP and TAZ (details given in the main text). In the cytosol, YAP/TAZ are phosphorylated (P) and are bound by proteins from the 14-3-3 family, which prevent their translocation into the nucleus. YAP can additionally be bound by angiomotin (AMOT). Dephosphorylation of YAP/TAZ is mediated through regulators as diverse as focal adhesion kinase (FAK), cellular sarcoma (Src), coiled-coil-containing protein kinase 1 (ROCK), G-protein coupled receptors (GPCR), α-actinin, or the junctional proteins neurofibromatosis 2 (NF), KIBRA, and Salvador-homologue 1 (Sav1). RhoA also interacts with TAZ. In the nucleus, YAP may be trapped by zona occludens 2 (ZO-2) protein. Otherwise, YAP/TAZ interact with TEA domain family (TEAD) transcription factors to regulate gene expression. Genes written in green, such as cysteine-rich angiogenic inducer 61 (*CYR61*), connective tissue growth factor (*CTGF*), runt-related transcription factor 2 (*RUNX2*), osterix (*OSX*), osteopontin (*OPN*), *ARMUS*, collagen 1 (*COL1*), α-smooth muscle actin (α-*SMA*), cellular myelocytomatosis (*c-myc*), and cyclin D1 are upregulated by YAP/TAZ. Contrary to that, the pro-apoptotic B-cell lymphoma 2 (*Bcl-2*) as well as cyclin-dependent kinase inhibitor (*CDKI*) transcripts are downregulated by these transcriptional co-activators (yellow). Details are given in the main text.

Angiomotin (AMOT) is a family member of the angiostatin-binding proteins and was described to bind to F-actin in the cytoplasm [123,124]. Upon the depolymerization of the cytoskeleton, which can be the result of changes in MT, AMOT dissociates from its binding partner and traps YAP/TAZ in the cytoplasm [125]. It is also a matter of debate if some unknown serine kinases may additionally be able to change the phosphorylation status of YAP/TAZ [126]. In the direct MT context, ROCK, FAK and Src have been described as important upstream regulators of YAP activity (Figure 2) [127,128]. The exact mechanism of how these proteins can interfere with the cytoplasmic-nuclear shuttling of YAP/TAZ remains to be elucidated, but indirect dephosphorylation through phosphatases such as PPM1A appears likely [129,130]. Similar to the bidirectional signaling of FAs, recent experimental evidence in human mesenchymal stem cells (hMSCs) also shows that YAP is not only regulated by FAK, but that FAK activity, as well as the protein abundancy of FAK and other FA components, is influenced by the cellular sublocalization and activity of YAP [61]. This leads to the conclusion that not only nuclear YAP exerts mechanobiological gene-regulatory functions, but that cytoplasmic YAP is also involved in MT by feeding back on FA activity and integrity. Additionally, the destruction of AJs by inhibiting the interaction of E-Cad with α-actinin also favors nuclear translocation of YAP. The latter mechanism was for example described in the context of enamel knot formation during tooth development [131]. Therefore, FAs as well as AJs are input signals for YAP/TAZ activity, which underscores their role as mechanoresponsive co-transcriptional activators.

Of interest, the transcription factor megakaryoblastic acute leukemia factor-1 and 3 (MKL1/3) is also translocated into the nucleus in a MT-related, force-responsive manner. Mechanistically, MKL1/3 binds go G-actin in the cytoplasm and dissociates from its binding partner as soon as G-actin is incorporated into growing actin filaments during polymerization. Apart from the AMOT-regulated YAP/TAZ transition, a similar mechanism might also apply to YAP/TAZ [132]. This elegant interdependency of YAP/TAZ and the actin cytoskeleton would effortlessly explain the mechanoresponsive features of these co-transcriptional regulators.

Within the nucleus, YAP/TAZ have to interact with transcription factors to regulate gene expression. The TEA domain family (TEAD) of transcription factors are the most important nuclear binding partners of YAP/TAZ. DNA binding of the YAP/TAZ-TEAD complex leads to the expression of core proliferation factors such as cellular myelocytomatosis (c-myc) or Cyclin D1 [133,134]. However, regulatory mechanisms to inhibit YAP/TAZ function also exist in the nucleus. The tight junction component zona occludens 2 (ZO-2), a protein also involved in cell-to-cell adhesion, can enter the nucleus and bind dephosphorylated YAP there. This shows, again, the tight interrelationship between “junctional” information and YAP/TAZ activity as well as the diverse signals that converge on these proteins [135,136,137].

Regarding the periodontium, YAP/TAZ is a central regulator in tooth and craniofacial development. Amongst others, the patterning genes of the Hox cluster are addressed by YAP/TAZ activity and the former’s function determines oral epithelial proliferation and the patterning of the enamel knot [138]. The formation of the cranial neural crest also depends on YAP/TAZ function and the induction of Fox transcription factors. The latter point is especially interesting in the context of periodontal regeneration as, e.g., stem cells in the PDL are neural crest derivates [139,140].

Specific findings in PDL stem cells (PDLCs) proved that cyclic mechanical stimulation results in an increase in nuclear YAP abundancy. Consequently, transcriptional YAP targets, such as cysteine-rich angiogenic inducer 61 (*CYR61*) and connective tissue growth factor (*CTGF*), are transcriptionally upregulated. By additionally inducing key differentiation markers of osteoblasts—amongst others, runt-related transcription factor 2 (*RUNX2*), osterix (*OSX*), osteopontin (*OPN*), and alkaline phosphatase—YAP is also involved in supporting the differentiation of PDLCs into osteoblasts (Figure 2) [141,142,143]. An orthodontic tooth movement (OTM) rat model as well as PDLCs in vitro experiments specifically proved that not only YAP but also TAZ supports osteogenic differentiation. The protein collagen triple helix repeat containing 1 (CTHRC1) was identified as a potent inductor of TAZ activity, which lead to an increase in collagen type 1, alkaline phosphatase, RUNX2, and osteocalcin [144]. However, YAP and TAZ seem to have slightly different biological functions during OTM. The immunochemical staining of histological sections from another rat OTM model pointed to the direction that TAZ activity might be more tightly coupled with *RUNX2* expression and thus osteogenesis, whereas YAP activity is more strongly connected to differentiation and proliferation [145,146]. This example demonstrates a common problem in YAP/TAZ research, namely that the cellular homologues are often regarded as equal, concerning their function. This assumption is, however, not justified. Another proof of a distinct regulatory mechanism of TAZ activity was described by Cui and colleagues. They reported that the micro-RNA miR-140 is involved in the regulation of osteogenesis of human PDLFs by interacting with a RhoA-TAZ signaling axis. In detail, the expression of this micro-RNA inhibits the transcription of RhoA, which leads to a reduction in TAZ protein activity in the nucleus and thus reduced osteogenesis during OTM. This is especially interesting as (i) this mechanism combines principles of transcriptional regulation on the RNA level with protein activity of important mechanotransducers, and (ii) was so far solely described for TAZ but not for YAP [147]. These slight differences might be decisive when it comes to dental implants and regenerative approaches with periodontal stem cells, as TAZ regulation and therefore cellular differentiation is sensitive to the environmental nano topography [148].

Apart from genuine periodontal tissues, YAP/TAZ were shown to be translocated into the nucleus in response to a static external magnetic field in human dental pulp stem cells and, thereby, induced mineralization beneath the cells. A concomitant rearrangement of the actin cytoskeleton was additionally reported [149]. This raises the general question of to which extent cells can respond to forces generated from static magnetic fields [150,151,152]. In the context of dentistry, it needs to be considered that ferromagnetic compounds can sometimes be found as part of a prosthetic material [153,154] within overdentures in the oral cavity of patients. As this material may potentially interfere with cell biology and MT, due to magnetism, and the exact molecular consequences are still a matter of scientific debate, it will be of great clinical interest to further study YAP’s involvement in these processes.

Concerning ECM homeostasis and regeneration, YAP promotes type I collagen synthesis. This finding together with the promotion of the osteoblast phenotype might explain the PDL-adjacent osteogenesis on the pull side during orthodontic treatment [142]. Alpha-smooth muscle actin (α-SMA) is supposedly also an indirect regulatory target of YAP/TAZ, as their nuclear presence was also shown to induce myo-fibroblast differentiation from PDLCs [155]. All these processes in PDLCs are, as already described, connected with actin-related cellular tension and thus MT. It is conceivable that a collagen 1-β1-integrin-RhoA-ROCK-F-actin-YAP/TAZ-collagen 1 signaling loop facilitates both the sensing and maintenance of the ECM and promotes PDLC lineage decision in a context-dependent manner [142].

Not surprisingly, YAP can maintain the stem cell properties of PDLCs in vivo and in vitro. When artificially overexpressed, YAP nuclear accumulation leads to an increase in proliferation, reduces cellular senescence and promotes apoptosis resistance [156]. On the molecular level, this is achieved via the upregulation of cyclin-dependent kinases (CDKs) and a downregulation of the latter’s inhibitors, the CDK inhibitors (CDKIs). Above, expression of B cell lymphoma 2 (Bcl-2) family members, as pro-apoptotic cellular regulators, is reduced (Figure 2). Moreover, mitogenic signaling, represented by ERK1/2 and MAPK/ERK kinase (MEK) activity, increases [156]. The proliferative capacity of an immortalized PDLCs-derived cell line, which was established via lentiviral transfection of human telomerase reverse transcriptase (hTERT), also depended on YAP nuclear activity. This can be shown by experimentally inhibiting YAP nuclear translocation via the photosensitizer Verteporfin [157,158]. Of interest, the cell line is not tumorigenic in nude mice, proving that hTERT expression in combination with high YAP nuclear activity alone is not sufficient for carcinogenesis [159,160]. Such experimental systems will be of great value for further periodontal research regarding appropriate approaches in regenerative medicine.

In the context of oral carcinogenesis, these stem-cell-like properties mediated by YAP bear the potential risk to support cellular transformation. Indeed, YAP overexpression was reported in many cancers, including OSCC [114,134,161]. Apart from the anti-apoptotic and proliferative activities, metabolic supply via the induction of autophagy as well as the regulation of EMT, are functions, which are at least partially attributed to YAP activity.

Recently, a novel mechanism of YAP-dependent OSCC proliferation was proposed. Piezo-type mechanosensitive ion channel component 1 (PIEZO1) is a mechanosensitive calcium channel and transcriptional target of YAP [162]. PIEZO1 signaling consequently leads to an increase in intracellular calcium concentrations, which promotes proliferation. This interesting insight into two mechanoresponsive proteins in the context of OSCC progression offers new perspectives in the pharmaceutical targeting of cancer cell-associated proliferative activity, by addressing the activity of PIEZO1 [163,164]. An up-to-date discussion of the Piezo channel family members and their role in mechanobiology was recently presented by Jiang and colleagues [165].

The increase in autophagic flux is mediated via the YAP/TAZ-dependent transcription of Armus, a Ras-related in brain (RAB)-GTPase activating protein (GAP)-family member, which is involved in maturation of autolysosomes from autophagosomes [166,167]. It is a matter of ongoing research to further clarify the interdependency of MT, autophagy, and metabolic integration, also with respect to periodontal tissues.

Regarding EMT, matrix stiffness is a key determinant of YAP activity. As also holding true for OSCC, tumor stroma is often regarded as desmoplastic, which goes along with an increase in the Young’s modulus and promotes nuclear YAP translocation [168]. β1-integrin-FAK-RhoA activity is also elevated under such conditions, as shown in a hepatocellular carcinoma (HCC) mouse model [169]. Nuclear YAP is then able to promote the switch from an epitheloid to a fibroblastoid cellular phenotype, as it controls both the activity and localization of Twist1. The latter, together with Slug and Snail, is a key transcription factor responsible for the molecular changes that occur during EMT, such as the downregulation of E-Cad and concomitant upregulation of fibroblastic marker proteins such as neural cadherin (N-Cad) or vimentin [170,171,172]. Taken together, it is conceivable that YAP is an important link between ageing, which is accompanied by an increase in ECM stiffness, and carcinogenesis related EMT. Regarding dental research, the clinical or diagnostic implications in the specific OSCC context remain to be elucidated.

Inflammatory periodontal diseases such as periodontitis are also linked to YAP and its role in mechanobiology. Especially the combination of occlusal trauma and periodontal inflammation seems to support periodontal destruction. As recently shown, the inhibition of YAP dephosphorylation and thus its nuclear translocation, could decrease inflammatory signaling in a mouse model of occlusal trauma and periodontitis. This shows the interrelationship of mechanobiological and inflammatory cellular pathways [173]. Alveolar bone loss was also linked to YAP activity and its interaction with Jun N-terminal kinase (JNK) and activator protein 1 (AP-1) [174].

Taken together, the described mechanisms of the action of YAP and TAZ in the periodontal context show both the complexity of the mechanobiological signaling integration and the decisive role of these processes in disease conditions such as periodontitis or OSCC (Figure 2) [175].

## 4. The Gist of the Matter: Nuclear Mechanotransduction

Section 3 discussed the mechanisms of YAP/TAZ dependent MT and its implications for periodontal tissues. As YAP/TAZ are soluble proteins, which shuttle between the cytosol and the nucleus, their mode of action can be described as a biochemical way of transmitting biophysical information. However, what about a higher physical intimacy between the cytoplasm and the nucleus?

In recent years, it has become more and more evident that nuclear shape and genomic integrity as well as gene regulation is directly linked to nuclear architecture and nuclear mechanotransduction (NMT) [26,176,177]. It is important to note that the molecular findings concerning NMT have been worked out in many different in vitro and in vivo model systems. Direct studies in cells from periodontal tissues are barely reported in the literature. As the proteins involved in NMT are highly conserved, it is, however, conceivable that the general mechanisms discussed below also apply to cells of periodontal tissues [178,179,180]. It is, therefore, a further research challenge to elucidate the cell-specific properties of NMT and their role for tissue homeostasis in the periodontium.

**Figure 3:**
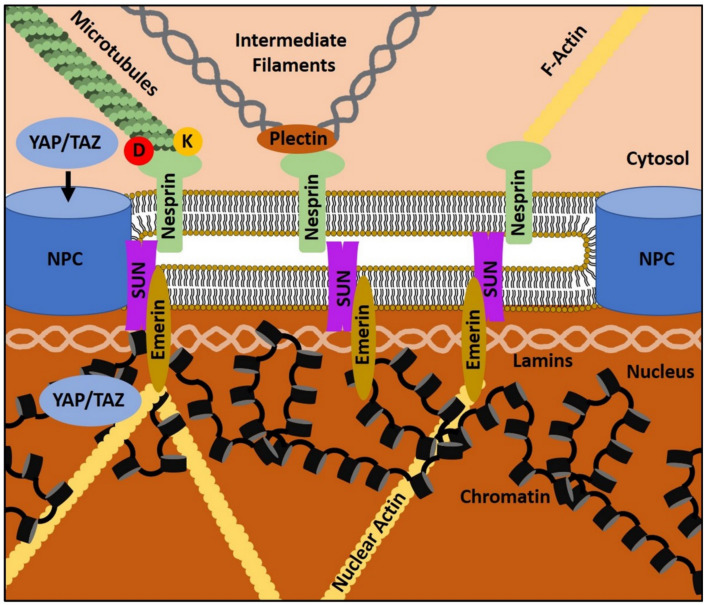
LINC complex-dependent mechanotransduction at the cytosol–nucleus interface. Nuclear mechanotransduction is a process at the cytosol–nucleus interface, where mechanobiological information is exchanged between the cyctosol and the nucleus and vice versa. The linker of nucleoskeleton and cytoskeleton (LINC) complex consists of Nesprins, which are embedded in the outer nuclear membrane, and Sad1p and UNC-84 homology (SUN) proteins in the inner nuclear membrane. At the nuclear periphery and within the nucleus, LINC is connected to nuclear pore complexes (NPC), Emerin, and the nuclear intermediate filament system, which consists of Lamins. This is the reason why LINC is directly and/or indirectly connected to the chromatin and the nuclear actin filament system. In the cytoplasm, Nesprin interacts with all cytoskeletal systems. Microtubules interact with Nesprins through the motor proteins Kinesin (K) and Dynein (D); intermediate filaments are connected to Nesprins via Plectin. Filamentous actin (F-actin) can directly bind Nesprins. Mechanoresponsive translocation of YAP/TAZ through NPCs into the nucleus is also connected to nuclear mechanotransduction (NMT). Details are described in the main text.

Various proteins that are localized in the outer nuclear membrane (ONM), the perinuclear space (PNS), the inner nuclear membrane (INM), or the nucleoplasm are directly or indirectly linked to mechanobiological functions and can, in many ways, be regarded as the nuclear equivalent of FAs [26]. From this perspective, the cytosol, similar to the ECM, represents the extranuclear environment in which cytoskeletal structures depict adhesion points for nuclear receptors. The most important structure that mechanically connects the cytoplasm with the nucleus is the linker of the nucleoskeleton and cytoskeleton (LINC) complex. LINC consists of members of the Klarsicht, ANC-1, and Syne-homology (KASH) domain protein family and members of the Sad1p and UNC-84 homology (SUN) protein family [181,182]. The former consists of Nesprin 1-4 in humans, which are embedded in the ONM. Of interests, Nesprins can physically interact with (i) plectin, a binding partner of intermediate filaments (IFs), (ii) kinesin and dynein, which are the motor proteins of microtubules, and (iii) directly with F-actin [183,184,185,186,187]. This means, that Nesprins can get in contact with all kinds of cytosolic cytoskeletal filament systems, rendering them the perfect adhesion proteins for transmitting cytoskeletal tension, including actomyosin contractility, into the nucleus. Nesprins further bind SUN proteins, namely Sun1 and Sun2, which span the PNS and the INM. Within the nucleoplasm, SUN proteins are in direct physical contact with a network of Lamins, nuclear pore complexes (NPC), and chromatin (Figure 3) [26,188].

Lamins are IFs in the nucleus and are encoded by three genes in men: *LMNA, LMNB1* and *LMNB2*. *LMNA* encodes for the two protein isoforms, Lamin A and Lamin C. Lamin B1 and Lamin B2 are the corresponding gene products of *LMNB1* and *LMNB2*, respectively [189,190]. All Lamin types form a tight meshwork of filaments of approximately 10–30 nm thickness along the INM [191]. Above, Lamins directly interact with chromatin, transcription factors, and proteins, such as Emerin [192]. The functions of the respective Lamin isoforms are interdependent and only partially redundant, as the deletion of one isoform leads to a loosening of the whole meshwork [26,193]. NPC localization is also associated with Lamin function. As the NPC is the central gate for the nuclear-cytoplasmic shuttling of proteins and vice versa, Lamins indirectly influence the exchange of soluble factors between both compartments [191,194]. Forces that lead to nucleus deformation, such as during cellular spreading on 2D culture substrates, result in a mechanical opening of NPCs and facilitate the nuclear entry of YAP [195]. However, sole osmotic nuclear swelling does not lead to the same result [195]. This elegant mechanism shows the stringent regulation of NPC opening and its relation to mechanobiology.

Besides IFs, the nucleus also harbors actin and actin-binding proteins. G-actin has been shown to act as a co-transcription factor for RNA-Polymerases 1−3, and to modulate the activity of other transcription factors and epigenetic regulators, such as histone deacetylases [196,197,198,199,200]. Upon polymerization, nuclear G-actin can form nuclear actin filaments. This process is triggered by cell spreading or adhesion and seems to correlate with the activity of integrins, cytosolic actin and the LINC complex [201]. Emerin, as well as Lamin A and B, have also been shown to bind nuclear actin [202,203,204]. Similar to its cytosolic analogue, nuclear F-actin is involved in the maintenance of nuclear integrity and shape and even participates in the cell’s stress response during replication [205]. Consequently, coordinated gene expression and genomic integrity is not only a result of biochemical factors and their related signal transduction, but is also based on nucleo-cytoskeletal dynamics and NMT.

The molecular architecture of the LINC complex in conjunction with its associated molecules in the cytoplasm and the nucleus reveals how biophysical cues can be mechanically transmitted from the cytosol to the nucleus and back. Exemplarily, integrin-transmitted forces can lead to changes in cytosolic actomyosin contractility, which is further transduced into the nucleus via LINC. LINC addresses lamins, nuclear actin and chromatin regulating proteins, and thereby regulates the condensation of chromatin, i.e., the transition between eu- or heterochromatin, which changes the cell’s expression profile and promotes cellular adaptation to the initial extracellular cue [26]. The reverse process; namely, the transduction of nuclear mechanical properties, such as stiffness, to the cytoplasm and consequently to the ECM via integrins is less well studied. Nonetheless, there is unambiguous evidence that LINC-dependent NMT is no one-way road. A stiffer nucleus facilitates FA formation and the newly built FAs are larger [206]. As above, the deletion of Nesprin-1 impairs the cell’s ability to adequately adapt to cyclic strain. This means that the nucleus is directly involved in the regulation of cytoplasmic actomyosin contractility, which consequently feeds back on FAs and the ECM through integrins [207]. Additionally, the nuclear position within the cell changes during these NMT processes. To prevent nuclear damage, the whole nucleus is oriented perpendicular to the stretch vector and parallels actin stress fibers [208]. This extends the above-discussed ECM-cytosolic mechanosignaling network to a bidirectional ECM–cytosol–nucleus network that is relevant to MT. Similar to cells as a whole, the nucleus can, therefore, also be characterized by physical properties, such as compressibility and elasticity [25,209].

Aside from the cytoskeletal coupling, described above, mechanosignaling-inherent post-translational modifications, together with conformational changes in involved molecules, also influence NMT. Lamin A and C, as well as Emerin, are subjected to phosphorylation in response to mechanical cues, such as changes in culture substrate stiffness. Emerin phosphorylation is mediated by Src, which is an FAs component and other kinases such as Abelson murine leukamie viral oncogene homolog 1 (Abl1) [210,211,212]. Lamin was reported to undergo conformational changes in response to mechanical loading, which might uncover the actual phosphorylation site [213]. This switch- or spring-like behavior has also been shown for α-catenin, where mechanical stretching exposes the vinculin binding site of α-catenin [214]. The phosphorylation is also a mean to mediate nuclear stiffness and cytosolic-nuclear coupling as well as regulation of YAP/TAZ nuclear abundancy. This indirect regulation of YAP nuclear import might explain why certain Lamin mutations lead to unphysiologically high levels of nuclear YAP [215].

Chromatin structure and dynamics change in response to mechanical stimulation, which is a result of the integration of the above-described signaling processes. Depending on the intensity and duration of the stimulus, the exact type of chromatin change varies [216]. On the short time scale, a reduction in histone methylation and thus heterochromatin leads to a nuclear softening. This means that the genome is protected from damage by decoupling the chromatin from the lamina until adaptation processes, such as cellular reorientation, have been accomplished [217]. Long-term effects include adaptations in methylation patterns and thus epigenetic changes. For example, an increase in H3K27me3 (tri-methylation on Lysine residue 27 of Histone H3) can be detected, which is a marker of heterochromatin and therefore gene downregulation [218]. Conversely, it is unclear how specific genuine mechanosensitive genes are switched on in response to mechanical loading, as so far experimental results are only available from artificial gene constructs [219]. It will thus be of great interest to analyze chromatin structure and dynamics in mechanically stimulated periodontal tissues and to see, which cell-type specific changes in cell behavior and gene expression can be attributed to NMT and related mechanisms.

As stated at the beginning, NMT concepts have, so far, been barely worked out in a periodontal context. However, morphological findings give indirect evidence that cells such as PDLFs also strictly depend on NMT and mechanical nuclear integrity. In one such study, an interesting link between ATP levels, ATP receptors and cell shape has been made. According to these findings, extracellular ATP levels are upregulated upon mechanical loading and nuclear deformation. The ATP-associated signaling cascades result in an increase in the receptor activator of nuclear factor kappa-B ligand (RANK-L) expression in PDLFs, which contributes to alveolar bone resorption. The latter process, which is important for periodontal regeneration disease, might, therefore, be in part the macroscopic correlate of periodontal NMT [220].

During tooth eruption, the PDL undergoes dramatic morphological and mechanical changes. The Notch pathway is involved in this process and responds to cell-to-cell and cell-to-matrix mechanical cues. Of interest, a transcriptional downstream target of mechanically activated Notch signaling in the PDL is Lamin A, whose Notch signaling-derived increase in expression supports the notion of the involvement of NMT in the periodontium [221].

In a broader medical context, it has been recognized for decades that mutations or changes in nuclear Lamins can have dramatic consequences for the individual. There exists a disease group called laminopathies, which are all related to aberrant functions of Lamins [222]. Envelopathies additionally include the disease of Lamin-associated proteins, such as Emerin or Nesprin [223]. The most relevant medical syndromes are Charcot–Marie–Tooth neuropathies, Emery–Dreifuss muscular dystrophies, and Hutchinson-Gilford syndrome, which is also known as Progeria [224,225,226]. Mechanically exposed tissues, such as the skeletal muscle, cardiomyocytes and tendons, are often affected by these diseases [26].

Two different hypotheses in the current literature discuss the reasons of the deleterious effects of the laminopathies. The gene regulation hypothesis claims that the mutations or defects in LINC components or Lamin/Emerin lead to a severe dysregulation of gene expression [227]. Consequently, cellular physiology is dysregulated, impairing, e.g., stem cell differentiation, and thus resulting in the clinical disease manifestations such as premature aging or muscle weakness [228]. Contrary to this, the structural hypothesis states that nuclear deformation and fragility caused by the mutations is the most important step in the disease process. However, a more holistic “MT-hypothesis” of the pathophysiology should actually incorporate both the gene dysregulation and the mechanobiological consequences, as both are highly interdependent [229]. This can be explained by the above-discussed functions of LINC and Lamins. The mutations associated with envelopathies are, therefore, valuable models for elucidating both general and tissue-specific mechanisms of NMT and its integration into the cellular context. One interesting molecular insight is the fact that certain Lamin mutations increase the abundance of phosphorylated Lamin. This subsequently increases the solubility of the protein and promotes its dissociation from the Lamin meshwork and thus creates nuclear fragility [230,231]. Additionally, direct DNA damage and cell-cycle arrest are associated with these mutations [232]. Reduction in the function of the ATPase associated with diverse cellular activities (AAA+ ATPase) TorsinA or rather its *Caenorhabditis elegans* ortholog OOC-5 have been shown to rescue the Lamin mutation phenotype [233]. This is interesting, as TorsinA normally enhances NMT through LINC regulation, and mutations in the gene are associated with dystonia and joint contracture [234,235]. Thus, in the presence of a mutated and, therefore, dysfunctional Lamin, an additional decrease in TorsinA activity with concomitant reduction in NMT prevents nuclear damage. This means that, under certain circumstances, the decoupling of cytoskeletal and nuclear mechanics is favorable for cell survival [236].

In the context of dentistry, delayed tooth eruption and impairments in dental root integrity as well as micrognathia are also related to Lamin mutations [237]. Patients suffering from Hutchinson–Gilford–Syndrome sometimes show developmental abnormalities in the craniofacial region and suffer from early onset periodontitis [238]. Based on the discussion of NMT, we hypothesize that mechanobiological ECM–cytosol–nucleus networks are in such a manner defective that both embryological processes as well as host–oral microbiome interactions are fundamentally misguided. Testing this hypothesis experimentally will shed light into both the pathophysiological mechanisms of envelopathies and their direct consequences for periodontal tissues.

Taken together, LINC-dependent NMT, Lamins and nuclear actin are fascinating aspects of an integrated ECM-cytosol-nucleus signaling hub, which orchestrates mechanobiological signaling pathways. Mechanically exposed tissues are particularly dependent on functional coupling of mechanical processes in the cytosol and the nucleus and dysfunctions in this system lead to severe diseases, such as laminopathies.

## 5. *Porphyromonas gingivalis*-Derived Proteases: A “Heavy Load” for the Periodontium

Having discussed the intrinsic properties and functions of cellular MT and NMT, it is important to have a look at exogenic factors interfering with core mechanobiological processes, which thereby affect periodontal tissue homeostasis. Approximately 20–50% of the worldwide population is affected by periodontal disease. In particular, periodontitis, an inflammatory condition thought to also support systemic cardiovascular and neurodegenerative diseases, as well as diabetes, leads to deleterious consequences such as loss of teeth [239,240,241].

The exact pathogenesis of periodontitis, as well as the onset of gingivitis and its shift towards periodontitis, are still incompletely understood. Many different models have been proposed in the last few years that try to incorporate the host’s individual susceptibility to periodontal disease, the immune system’s response to bacterial invasion, as well as bacterial colonization and virulence factors [242,243,244]. Above, it is still a matter of debate if bacterial infection is causal for periodontitis and if it really precedes the host’s inflammatory response. Apart from that, it is, however, clear that changes in the composition of the bacterial population, the oral bacterial load, and the oral milieu can promote oral dysbiosis. The interindividual presence and variance of different bacterial taxa in the subgingival region under healthy and pathological conditions has, therefore, been intensively studied during the last few years with the help of, e.g., next-generation sequencing (NGS) techniques [245,246].

The subgingival microbiome harbors around 500 different bacterial species, while a few dominate under healthy conditions. Among them, the Gram-positive bacteria *Actinomyces naeslundi*, *Actinomyces meyeri*, *Rothia aeria*, *Rothia dentocariosa*, *Streptococcus sanguinis*, *Streptococcus oralis*, and *Streptococcus intermediuas* are commonly found in great quantities. The Gram-negative bacterium *Fusobacterium nucleatum* also contributes to the composition of the normal subgingival plaque [247,248]. The above-described aetiologic factors can, however, induce qualitative and quantitative shifts in the oral microbiome, which contribute to periodontal inflammation and subsequently periodontitis. This process mostly functions with an increase in Gram-negative species, especially the bacteria from the so-called “red complex” (originally described by Sigmund Socransky), namely *P. gingivalis*, *Treponema denticola*, and *Tannerella forsythia*, emerge and play a pivotal role in periodontitis. Moreover, a strong association of oral dysbiosis and periodontitis with *Aggregatibacter actinomycetemcomitans*, *Bacteroides spp.*, *Fretibacterium spp.*, *Desulfobulus spp.*, and *Parvimonas micra* is reported in the literature [249,250]. All of those periodontopathogens lead to a mixed infection of the periodontium and directly contribute to periodontitis via virulence factors or indirectly through immunopathology.

Host factors contributing to periodontitis include various chemokines, pro-inflammatory cytokines, and MMPs, as well as arachidonic acid derivates such as Leukotriene B4 and Prostaglandin E2 [242,251]. Apart from that, the serum-like composition of the crevicular fluid specifically promotes the growth of protease-rich taxa, which stimulate the progression of destructive processes during periodontitis [252,253]. These proteases are able to destroy the host’s protease inhibitors, such as secretory leukocyte protease inhibitor (SLPI), which further contributes to disease progression [254]. The actual inflammation consequently arises from the complex spatio-temporal interplay of these host factors with the subgingival microbiome and its virulence factors.

Among the many bacteria found in the subgingival region of periodontitis patients, the anaerobe, Gram-negative bacterium, *P. gingivalis,* has been extensively studied and is seen as one major etiologic factor in severe periodontitis [255]. This is the reason why its virulence factors and their interaction with MT are currently best understood compared to the above-mentioned pathogens. Therefore, this section focuses on the intricate interrelationship between periodontal MT and *P. gingivalis*.

Aside from lipopolysaccharides (LPS), the bacterial capsule and fimbriae, *P. gingivalis,* expresses another group of virulence factors, named gingipains [256]. The latter are cysteine proteases with a specificity for arginine (HRgpA and RgpB) or lysine (Kgp) and are either secreted or non-covalently attached to the bacteria. Molecular studies over the past few years have shed light into the exact functions of gingipains during the initiation and promotion of periodontitis. Of interest, these proteases not only induce and modulate pro-inflammatory cytokines but also digest host antibodies and are involved in hemagglutination [257].

From a mechanobiological point of view, gingipain proteases serve as an important example of how external pathogen-derived virulence factors can interfere with MT pathways by either interacting with or destroying its signaling components. Therefore, without generating genuine mechanical signals, *P. gingivalis* metaphorically imposes “heavy loads” on the periodontium via different mechanisms.

Concerning the ECM, gingipains have been shown to degrade various collagen isoforms, including collagen type I, III, IV and V, as well as fibronectin (FN). Interestingly, HRgpA and Kgp possess hemagglutinin domains, which directly guide the proteases to FN [258]. FN degradation generates FN fragments, which can be used as a periodontitis biomarker in the crevicular fluid of patients [258]. These fragments also change the cell’s perception of their environment, as integrin-related signaling changes upon the recognition of such fragments [259]. Moreover, the proteases induce the expression of MMPs in tissue-resident fibroblasts, which further promotes catabolic ECM processes [260].

**Figure 4:**
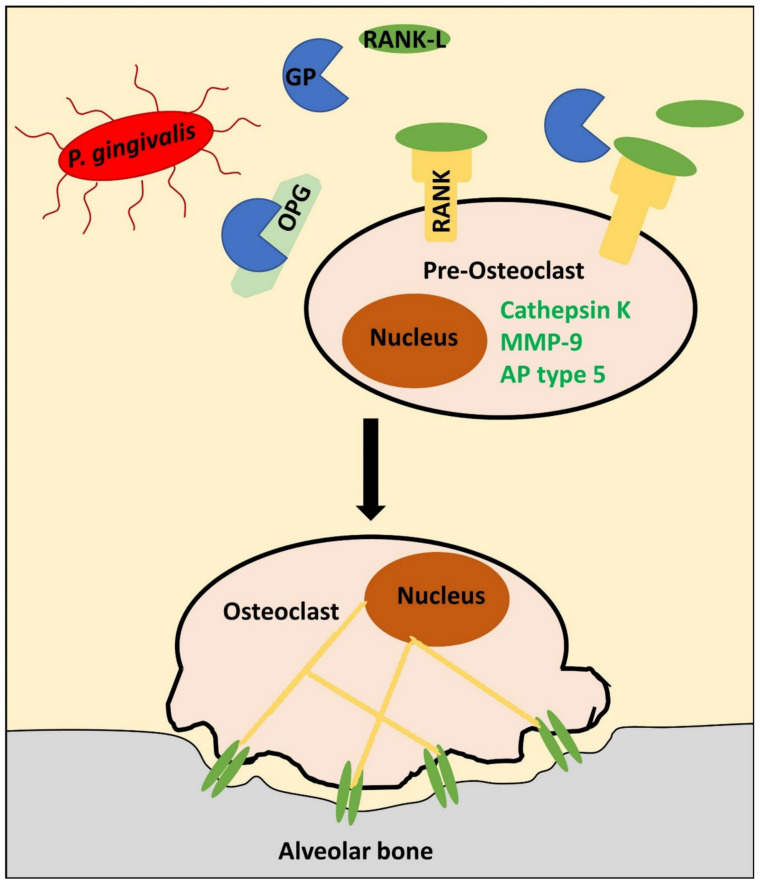
*P. gingivalis* induces alveolar bone resorption through gingipain proteases. Secreted proteases, so-called gingipains (GPs), from the microbe *Porphyromonas gingivalis* (*P. gingivalis*) interact with the receptor activator of nuclear factor kappa-B ligand (RANK-L), osteoprotegerin (OPG), and receptor activator of nuclear factor kappa-B (RANK) system of osteocytes and osteoclasts. In the presence of GPs, OPG is degraded, which favors RANK-L binding to RANK. Consequently, osteoclasts differentiate out of pre-osteoclasts through upregulation of Cathepsin K, matrix metalloproteinase 9 (MMP-9), and alkaline phosphatase type 5 (AP). Subsequently, osteoclasts can bind to the alveolar bone via αVβ3 integrins (green, dimeric sticks), which regulate actin cytoskeletal tension (yellow lines). Altogether, these processes favor alveolar bone resorption within resorption pits. Details are given in the main text.

Gingipain-related ECM degradation is, however, not limited to fibrous components, as these proteases, in conjunction with the receptor activator of nuclear factor kappa-B ligand (RANK-L), promote osteoclastogenesis, which is a prerequisite for the destruction of the mineral matrix during alveolar bone resorption in periodontitis [261]. In detail, gingipains induce osteoclast-specific genes, such as cathepsin K, MMP-9, and alkaline phosphatase type 5, in a dose-dependent manner [262]. This happens only in the presence of RANK-L, the ligand of the receptor activator of nuclear factor kappa-B (RANK) expressed in osteoclastic progenitor cells. RANK-dependent signaling is a strong inducer of osteoclastic phenotype and osteoclast function. In vivo, RANK-L is secreted by osteoblasts and its action can be perturbed via osteoprotegerin (OPG). The latter functions as a decoy receptor of RANK and is digested by gingipains, who thereby further support osteoclast activation by shifting the RANK-L to OPG ratio towards RANK-L [263,264]. Periodontal disease also leads to round PDLF nuclear morphology in vivo, which is accompanied by a reduction in actomyosin contractility and a decrease in OPG synthesis from PDLFs. This additional mechanism might, therefore, contribute to this shift in biochemical signaling and is a direct consequence of disturbed MT (Figure 4) [265].

Intracellularly, RANK downstream signaling activates inflammatory pathways such as nuclear factor kappa B (NF-κB) and nuclear factor of activated T cell c1 (NFATc1) signaling. Subsequently, the expression of β3 integrin is upregulated, whereas β5 integrin is suppressed [266]. The former is necessary for the building of αVβ3, which mediates the adhesion of osteoclasts to the bony matrix and enables the formation of resorption pits for matrix degradation. During this adhesion process, FAK and paxillin phosphorylation and activity increases, indicating active mechanotransduction between focal adhesion structures and the cytoskeleton. Taken together, gingipains promote MT cascades in the context of alveolar bone resorption by both facilitating osteoclast differentiation and integrin-dependent resorption pit formation [261]. It remains to be elucidated how the presence of gingipains exactly augments the function of RANK-L.

Findings from other studies suggest that certain integrins are not only indirectly targeted by gingipains through transcriptional regulation, but directly cleaved by the proteases. Hydrolysis of β1, α2 and α5 integrin is repeatedly reported in the literature. This leads to a loss of cell adhesions, as described for GKs, GFs, and osteoblasts. When a cell loses its contacts with neighboring cells or the ECM, cytoskeletal changes induce a rounding of the cell culminating in a specific form of apoptosis known as anoikis [267,268]. β1 integrin degradation was also shown to result in a remarkable decrease in cytoplasmic RhoA activity but not RhoA abundancy in an osteoblast cell culture system. As RhoA is an important regulator of actin cytoskeletal tension, the gingipain-induced events consequently led to F-actin disruption and, therefore, cell shrinkage or apoptosis. Overexpression of β1 integrin or gingipain inhibition could rescue this phenotype, sustaining the conclusion of a direct relationship between β1 degradation, cellular MT, and cell death. Apart from the above-discussed activation of osteoclast activity, *P. gingivalis* can further contribute to apoptotic bone loss in periodontitis [269].

Beneath extracellular catabolic processes, *P. gingivalis* is known to directly invade cells such as GKs. Therefore, gingipains can also trigger intracellular processes related to MT [270]. Kinane and colleagues reported that cytosolically released gingipains, especially the lysine-specific form, hydrolyze actin and provoke the collapse of the cytoskeleton and, consequently, apoptosis. This proves that the actin cytoskeleton, as the signaling hub of both outside-in and inside-out MT, is vital for cells. Moreover, the bacterial proteases downregulate caspase 3 activity, making it plausible that cell death occurs independently of caspases [271]. A similar observation has been made for endothelial cells [272].

AJ-dependent cell-to-cell adhesion is also affected by *P. gingivalis* proteases. Generally, E-Cad-mediated cell contacts, as well as tight junctions and desmosomes, are involved in epithelial barrier function by physically defining different physiological milieus and polarized cellular structures, such as apical-basal polarity. In GKs, this function is particularly important to protect other periodontal structures from invasion of the oral microbiome. GK and GF microtissue 3D models have clearly demonstrated that *P. gingivalis* invades the connective tissue of the gingiva by bypassing the epithelial barrier. This occurs alongide cytoskeletal changes and disruption of adhesion structures, thereby feeding back on MT processes [273]. E-Cad is also directly cleaved by Kgp. Loss of the extracellular cadherin domain disrupts the epithelial integrity of the oral mucosa [257]. *Porphyromonas* LPS additionally promotes transcriptional downregulation of E-Cad and thereby enhances gingival epithelial permeability [274]. Unsurprisingly, these processes go along with inflammatory responses, as can be seen from increases in reactive oxygen species (ROS) and tumor necrosis factor α (TNF-α) levels. Together with the recruitment of immune cells, this substantially contributes to periodontal destruction in periodontitis [105]. This subject is also relevant regarding dental implants. As was shown for titanium–zirconium alloys, gingipain-mediated E-Cad degradation prevents GK adhesion to the implants, which hinders proper material incorporation into the dental alveolus [275].

In the context of oral carcinogenesis, the interplay of E-Cad and *P. gingivalis,* as well as other periodontal pathogens, is also relevant, although the direct involvement of the gingipain proteases has not, so far, been shown. However, GK cell culture models clearly show that the pathogens promote epithelial to mesenchymal transition (EMT), a core step in carcinogenesis, by the downregulation of E-Cad. This accounts for the concomitant increase in neural (N)-Cad, vimentin, the transcription factor snail, as well as MMP-2, which are markers of a fibroblastoid phenotype and accordingly characterize the process of a phenotypic switch seen in malignant transformation. These observations are paralleled by an increase in the migratory ability of the GKs and hint at a potential invasive phenotype [276,277,278]. It is tempting to speculate that these phenomena at least in part depend on gingipain-mediated proteolytic processes of surface adhesion receptors. OSCC invasion is also promoted by an increase in MMP-9 in response to gingipain expression in the host. MMP-9 related ECM degradation inevitably feeds back to MT pathways and, hence, cell behavior.

From a regenerative perspective, microbial colonization of the oral cavity can also be protective. The germ *Akkermansia muciniphilia* was recently described to counteract protease-related damage of *P. gingivalis* by both sustaining an anti-inflammatory milieu through interleukin 10 (IL-10) and by enhancing the transcription of E-Cad and β1-integrin. Probiotics are, therefore, a tempting option in the treatment of bacteria-induced periodontitis [279].

Taken together, the discussion of *P. gingivalis*- and gingipain-related changes in mechanobiologically relevant signaling axes underscores the complex interplay of cells and their ECM and the diverse mechanisms of action occurring during periodontitis.

## 6. May the Force Be with You: MT and Its Implications for Periodontal Regeneration

In the context of MT, the previous four sections discussed the molecular principles of cellular mechanoperception and mechanotransmission, which are perspectively mandatory for any kind of force/MT-related periodontal regeneration approach. As can be seen by the enormous number of proteins involved and the extensive signaling crosstalk, it is evident that artificially modulating MT pathways can lead to unexpected results [13,280]. It is therefore of great importance to note that broader basic research is needed to elucidate cell-type specific and spatiotemporal aspects of MT, its exact regulation during the lifespan of an individual, and the dysregulation in pathological processes [281].

So far, there exists no therapeutic application in the context of oral regeneration and health that directly addresses MT pathways in order to enhance treatment efficiency. Nonetheless, it is worth discussing selected experimental strategies and rather theoretical concepts, which could in the future be used to support regeneration or to mitigate periodontal disease processes in patients by means of mechanobiological principles.

Regarding regenerative medicine, the development of innovative, mechano-active biomaterials plays an increasingly important role in the field of bioengineering with the ultimate goal to replace or imitate natural periodontal tissue environments [282,283]. Thus, ECM-mimetic, biocompatible substrates, such as hydrogels, are designed to initiate or maintain cell differentiation, proliferation or migration and should ideally lead to a restitutio ad integrum [284]. The increasing notion of the intricate role of periodontal ECM, adhesion structures, as well as the cyto- and nucleoskeleton as biochemical and biomechanical signaling platforms that govern cell behavior, makes it tempting to speculate that the proper integration of inductive and, therefore, cell-instructive stimulatory signals will allow researchers and clinicians to offer personalized therapeutic options to their patients in the future.

However, mechanobiological studies are often conducted as in vitro experiments with only a single cell type or with isolated tissue samples [146,285,286]. This leads to various difficulties in interpreting the results and in transferring them to the in vivo situation. It was shown by different groups that cell behavior is directly connected to their environment, meaning that culturing cells in 2D or 3D substrates makes an enormous difference [287]. Two-dimensional substrates, such as the classical polystyrene culture dishes, are, from a mechanobiological point of view, relatively simple, as they are easily characterized by stiffness (Young’s modulus), the material’s physiochemical properties (composition), and ligand density in the case of biofunctionalized materials [288,289]. Contrary to that, 3D materials offer adhesion points in all three dimensions and further show differences in porosity, microarchitecture, and local rigidity. This is the reason that processes, such as cell migration, differ between 2D and 3D substrates [290]. A higher substrate porosity also favors the migratory phenotype, which is especially important in studying pathologies like OSCC [291,292]. Above, interactions of different cell types and the role of the immune system and vasculature are seldomly addressed in mechanobiological studies [293,294,295]. These obstacles must be considered when discussing the application of MT principles regarding the design of, e.g., innovative dental implants for periodontal regeneration [296].

Considering integrin-dependent FAs, their roles in fibrillogenesis of ECM molecules, e.g., collagen or FN, wound healing, and osteocyte differentiation are particularly interesting for regenerative approaches. Consequently, an optimal intervention strategy should enable (i) tissue-specific ECM synthesis in response to integrin signaling, (ii) support and sustain the proliferation, differentiation, and migration of embedded and neighboring periodontal cells by also taking into account their developmental stage, (iii) inhibit matrix degradation, and (iv) sustain an overall anti-inflammatory oral milieu. Exemplarily, in the case of a periodontal defect or alveolar bone loss, a regenerative approach could either stimulate tissue-inherent stem cells in situ or make use of biomaterial-based strategies. The latter are specifically interesting, as such generally polymer-based materials can stimulate cells and guide their behavior by making use of biochemical and biomechanical signals. As above, (stem) cells can be directly incorporated into scaffold matrixes via bioprinting and transferred to the patient [297]. Besides, bioprinting offers the opportunity for tailored periodontal defect coverage in case of trauma or surgery. By considering all of these properties, such materials are sometimes designated to be “cell-instructive”, meaning that they harbor all necessary biochemical and biophysical information to properly integrate cells into the site of defect [298,299].

Guo and colleagues recently presented a modular platform of biodegradable crosslinkers for hydrogel engineering that can be adapted to tissue-specific needs. This crosslinker can be modified with small peptides or naturally occurring ECM components to specifically stimulate receptors on target cells. The amino acid motif arginine-glycine-aspartate (RGD) is important to bind integrins and can easily be incorporated into such synthetic hydrogels to stimulate integrin receptors and, thereby, FAs-linked MT pathways [300].

In order to selectively induce osteocyte differentiation from mesenchymal stem cells in the periodontium, such a stimulus alone is, however, not sufficient [301,302]. Additional spatiotemporal factors as well as biochemical factors need to be included [303]. Of note, biocompatible materials can be used as vehicles for drug delivery, which can for example be antibiotics or differentiation factors. In this context, spatiotemporal control of drug release is of enormous importance in order to induce and sustain the intended tissue phenotype by simultaneously avoiding unwanted off-target effects, such as systemic adverse effects, cellular toxicity, inflammation or the induction of malignant processes. Therefore, the cautious selection of drug delivering biomaterials under the consideration of tissue-specific conditions is obligatory [304]. In the periodontal context, this means that such systems should promote, e.g., bone or PDL regeneration from stem cells in the case of periodontitis-associated bone loss or mitigate inflammation in periodontitis through antimicrobial agents.

Generally, implantable drug delivery devices, such as exemplified by the below-mentioned injectable hydrogel, can be divided into passive polymeric implants or dynamic/active polymeric implants. The latter are mostly electronically regulated devices, such as pump type implants (e.g., for diabetes therapy) and are not discussed further here. The former can additionally be subdivided into non-biodegradable and biodegradable implants [304,305].

Non-Biodegradable implants are composed of synthetic polymers that are biocompatible but cannot be degraded within the body. Generally, these kinds of implants need to be removed after they have served their purpose. Silicones, poly(urethanes), and poly(acrylates) are commonly used for such implants. In the case of monolithic implants, the drug is homogeneously dispersed within the polymer, whereas reservoir-type implants contain a compact drug core with some sort of polymer membrane around it that controls diffusion and the release of the pharmacological compound [306,307,308,309].

Biodegradable polymers, such as poly(caprolactone), poly(lactic acid), or poly(lactic-co-glycolic acid) or naturally occurring poylmers, such as collagen or fibrin, can be degraded within the body. The latter process can be achieved via simple hydrolysis, enzyme degradation, redox reactions, or simply physical deterioration. The great advantage of biodegradable polymers is the fact that they can remain within the body, i.e., there is no need for removal [310,311,312,313].

Mechanistically, drug release in all these systems is either possible through controlled swelling (e.g., in hydrogels), osmotic pumping, or passive diffusion. Moreover, chemical triggers of drug release include pH-responsive sidechains or redox switches. Mechanically induced drug release through ultrasound, magnetic or electric fields, and temperature changes is also under investigation and these stimuli could work synergistically with tissue-inherent MT pathways. Matrix degradation is also possible in the case of biodegradable polymers, where either the polymer itself or biodegradable crosslinkers are digested and the drug is released thereby [304,314].

When considering drug delivery into a site of disease, it needs to be considered that the local micromilieu is different from physiological conditions. This means that different parameters, such as hypoxia, reactive oxygen species (ROS), blood vessel expansion, temperature, and acidity, need to be considered [314]. As discussed above for periodontitis in Section 5, the infection with a pathogen such as *P. gingivalis* can lead to the presence of proteases, which are normally not found within the human body.

For example, minimally invasive alveolar bone regeneration with injectable hydrogels offers a high degree of spatial and temporal control and are suitable for additional drug delivery. A recently presented thermosensitive hydrogel, which gelatinizes upon heating to 37 °C, made from β-glycerol phosphate, chitosan and collagen has been shown to support PDLCs growth. By adding osteo-inductive drug compounds, such an approach combines several stimulatory principles, thereby enhancing the bone regenerative capacity of the material [315]. Another recently published article presents a cellulose/κ-carrageenan oligosaccharid-based hydrogel with incorporated antimicrobial agents, which showed strong antibacterial activities in a periodontitis model [316]. As there is a vastly growing amount of such applications, the interested reader is referred to some recent, innovate research work, which cannot be discussed in detail here [317,318,319,320,321].

Synthetic integrin ligands, among them some that contain mutated RGD motifs, have also been shown to change MT in a mouse model [322,323]. It is, therefore, an interesting perspective to search for integrin-subtype specific ligands, which can selectively stimulate or inhibit integrins on different periodontal cell types and under different conditions, such as during periodontal wound healing or periodontitis. By exemplarily stimulating α5β1 integrin on PDLFs in the right context, collagen or FN fibrillogenesis could be enhanced to strengthen dentoalveolar integrity via MT pathways [324]. Khorolsuren, as well as Matsugami and colleagues, presented a study with PDLCs and such synthetic integrin ligands. Cell adhesion, as well as migration and osteogenic differentiation of the PDLCs, was promoted by different cyclic integrin-ligand mimics, thus proving the suitability of the concept [325,326].

Low-intensity pulsed ultrasound (LIPUS) is a method to mechanically stimulate tissues via ultrasound in the range of 1–4 MHz and 0.01−90 mW/cm^2^. LIPUS was shown to enhance ECM synthesis and regeneration and to be a potent stimulator of osteogenic differentiation and is used, e.g., in the context of fracture healing [327,328,329]. Bone mass and maturation is also increased upon LIPUS treatment in different animal models, and it was shown that osteocytes in particular respond to LIPUS treatment [330,331]. Transcriptional analysis in a mouse cell line revealed that approximately 180 genes from clusters related to transcription, cellular secretion and immunity are responsive to the mechanical stimulation [331].

Several studies have investigated the role of LIPUS in the periodontal context and it is a tempting mean to enhance oral regenerative approaches. Upon stimulation of PDLCs via LIPUS, osteogenesis in the alveolar region and ECM regeneration was observed [332]. Although not directly shown, it is conceivable that these adaptation processes are mediated through MT signaling pathways [333]. The endoplasmic reticulum unfolded protein response (ER-UPR) as well as autophagic pathways, as detected by an increase in LC3 and Beclin-1 expression, are molecularly involved in the regulation of PDLCs behavior under inflammatory conditions [328,334]. LIPUS also reduces oxidative stress [335] and downregulates the expression of pro-inflammatory cytokines such as IL-8 and inhibits the NF-κB signaling axis [336]. This is of clinical interest, since inflammation, as observed during periodontitis, normally inhibits osteogenesis. It is, however, not clear how the mechanical ultrasound waves are transmitted to induce these cellular signaling responses. A recent study on dento-alveolar integrity during orthodontic force application in normal and diabetic rats could show that LIPUS leads to a significant increase in predentin and cementum thickness in all study groups. As above, the number of odontoblasts, as well as periodontal ligament cells, increased. On the tension side, bone remodeling was enhanced, whereas the number of resorption pits increased on the compression side [337]. LIPUS also augments bone formation in the context of osseointegration of dental implants in mice [338]. As the molecular mode of action of LIPUS is still insufficiently understood, it is a further research challenge to determine if and to what extent the herein discussed MT pathways are involved in the above-described cellular responses to the treatment. It is conceivable to assume that different periodontal regenerative approaches could benefit from additional LIPUS application in order to increase therapeutic effects.

Receding gums are, likewise, a relatively common problem and, therefore, gingival regeneration is of high clinical interest [339]. Stratified epithelia, such as the gingival epithelium, show a complex pattern of differentiation markers in an apical-basal direction, such as different keratin isoforms or involucrin in the gingival epithelium [340,341]. In vitro experiments with electrospun, gelatin-based matrices with a specific elasticity of 3.2 kPa proved to promote proper gingival morphogenesis, which was independent of a co-culture with mesenchymal cells. Molecularly, this process depends on the ERK1/2-β1-integrin signaling axis, underlining the role of MT-triggering basal integrins for gingival epithelial tissue morphogenesis [342,343,344]. These promising results show that addressing MT through integrins by choosing a biocompatible material with the right stiffness is a cornerstone for tissue engineering and prospective regeneration of periodontal tissues, such as the gingiva.

The modulation of AJs-related signaling is important in the context of mucosal barrier function and OSCC suppression. As discussed in Section 1, Cads also sustain periodontal ECM and induce collagen 1 and elastin synthesis [345]. Cad-dependent signaling can be addressed and mimicked by the same principles as discussed above for integrins. Additionally, we hypothesize that the cleavage sites of gingipains within the E-Cad amino acid sequence could be an interesting target to be addressed by mutational studies. It is tempting to mutate the sequence of E-Cad, rendering it insensitive to gingipain digestion. Cell culture experiments and animal studies would need to support the idea of reducing destructive and inflammatory consequences of gingipains and the normalization of AJs-dependent MT by this approach. If successful, patients suffering from severe periodontitis could then be treated with autologous epithelial stem cells, expressing the protease-resistant E-Cad under the control of a constitutively active promotor in the long run [346,347]. Thus, in combination with pro-epitheliogenic biomaterials, gingival integrity could potentially be increased while simultaneously decreasing the chance of periodontitis associated OSCC development. This hypothesis is, of course, highly speculative, but should be considered by addressing it experimentally.

The indirect modulation of E-Cad expression and the activity of inflammatory cellular pathways in the periodontium is possible via vitamin D administration. Oh and colleagues showed that GKs respond to the vitamin by upregulating E-Cad and downregulation of NF-κB and MMPs. This offers the possibility to change the expression pattern of mechanobiologically relevant proteins by the simple administration of a widely available, single biochemical molecule [348]. Similarly, junctional epithelial function in a model of peri-implantitis was enhanced by application of a low molecular weight JNK inhibitor. Mechanistically, this promoted E-Cad upregulation and F-actin regulation [349]. Both examples show that biochemical modulation, apart from actual mechanical stimulation, is a promising alternative to influence MT through changing the expression patterns of its molecular constituents.

The actin cytoskeleton as the central hub of AJs, FAs, and NMT signaling is another interesting target to be addressed by intervention strategies for periodontal tissue regeneration [350,351]. The difference in addressing AJs or FAs on the cellular surface is that RhoA, Rac1, Cdc42 or ROCK cannot be modulated via direct interaction with a biomaterial. As these are all cytoplasmic proteins, low molecular weight pharmacological compounds or RNA-based strategies are promising candidates. A recently published proof-of-principle study addressed RhoA transcription via a drug-releasing polymer containing a RhoA-siRNA embedded in a nanocarrier [352]. Efficient drug delivery and subsequent downregulation of RhoA was shown, and this principle could also be used in periodontal tissues to modulate cytoskeletal stiffness during different phases of regeneration.

The well-known RhoA activator Calpeptin is another candidate to influence cytoskeletal MT processes. Contrary to siRNA, Calpeptin enhances the activity of RhoA and is able to increase actomyosin contractility and thus cell stiffness [353,354]. This is especially important in the context of alveolar bone osteogenesis, where increased ECM and cytoskeletal stiffness favors osteogenic differentiation patterns [355].

Furthermore, cell penetrating peptides (CPPs) offer the possibility to deliver cargos to the cell’s interior [356]. CPPs are covalently or non-covalently linked to small drugs, peptides, proteins, or nucleic acids, which are thereby delivered to the cell [357]. Thus, the small GTPases or modulators of their activity could be directly transported to the cell via CPPs. This regenerative strategy would be suitable for local and targeted therapy of periodontal defects without implanting a foreign material into the patient. First clinical trials with the application of CPPs in humans have been reported and have shown no severe toxic effects so far [358,359]. Therefore, this technology may be broadly available in the future. The applicability and effectiveness in oral tissues, however, remains to be determined.

YAP and TAZ have been implicated in many in the periodontium, including myofibroblast differentiation, osteogenesis, and oral mechanobiological carcinogenesis processes. Addressing these Hippo components pharmacologically is not a new concept, as their role in many different human cancers makes them a promising target to slow down disease progression or hopefully to suppresses features of malignant transformed tumor cells [164,360,361]. There exist different mechanisms of how to prevent YAP nuclear entry and thus induction of proliferation and EMT.

Verteporfin is a photosensitizing pharmakon, which is already in clinical use in ophthalmology [362]. Verteporfin enhances cytoplasmic 14-3-3σ, which in turn binds YAP and prevents its translocation into the nucleus [363]. Regarding the stiff, desmoplastic stroma in OSCC, Verteporfin-enhanced treatment strategies could help to reduce the stiffness-susceptible and thus MT-sensitive proliferative capacity and migratory potential of malignant cells.

Nuciferin is an alkaloid compound from lotus plants and was recently shown to sensitize cancer cells to chemotherapeutic agents [364]. Mechanistically, Nuciferin promotes adenosine monophosphate-activated protein kinase (AMPK)-related YAP phosphorylation on Ser127 and consequently cytoplasmic trapping of the co-transcriptional activator. It will, therefore, be interesting to see if similar principles apply to OSCC.

Sophisticated, innovative biomaterials have also been evaluated in the context of mechanobiology and YAP. As regenerative stem cell therapies have great therapeutic potential, it is still an active field of research, if such approaches in mechanically exposed tissues such as joints or the periodontium benefit from concomitant mechanical stimulation of the transplanted cells. The so-called “loading history” of the cells might, therefore, play an important role in regenerative stem cell therapies and underscore the pivotal role of MT [365,366,367]. To study these potential influences, Kojima and co-workers presented a micropatterned, polymer-based micropillar research platform that enables the study of FAK and YAP activity in response to mechanical loading. As a special feature, the skillful combination of two biocompatible polymers guarantees highly specific cell adhesion on the top surfaces of the micro-posts. The simultaneous integration of magnetic nanoparticles into the polymer additionally offers the possibility to magnetically actuate the pillars to exert shear forces on the cells [368]. The first biological data from human mesenchymal stem cells show that early adaptation of cells to cyclic mechanical loading includes the recruitment of phosphorylated and, thus, active FAK to the cellular periphery and the translocation of YAP into the nucleus [369], thereby potentially supporting regeneration-relevant cell proliferation or differentiation. It will be interesting to apply such mechanobiological test platforms to periodontal cells and even to try to develop the principle further to actual mechano-active, implantable biomaterials.

Concerning innovative biomaterials that have the potential to regulate YAP/TAZ activity, the technique of guided bone regeneration (GBR) offers great regenerative potentials. In the GBR of periodontal tissues, biocompatible materials are used as spacers between connective tissue and the alveolar bone in order to prevent overgrowth of gingival soft tissues by simultaneously allowing the regeneration of bone. This is especially important in periodontitis, where alveolar bone loss and hence the ensuing loss of teeth is a major problem [370,371].

Different materials and strategies for periodontal GBR have been proposed in recent years. Porrelli and colleagues presented an electrospun polycaprolactone-based biomaterial, which was functionalized with lactose-modified chitosan and antimicrobial silver nanoparticles. They could show that osteoblast adhesion and proliferation was significantly enhanced with this regenerative approach. As above, the incorporation of the antibacterial nanoparticles prevented biofilm formation of bacteria such as *Staphylococcus aureus* and *Pseudomonas aeruginosa*. Cytotoxic effects were not reported [372]. Another study by Balbinot and co-workers used a polybutylene adipate terephthalate biodegradable membrane, which was enhanced with niobium-containing bioactive glasses. Again, the material was permissive for osteogenesis [373]. GBR can also be enhanced via incorporation of pharmacologically active compounds. Recently, a thermosensitive Pluronic F127/poly(lactic acid) formulation with zoledronic acid nanoparticles was reported, which lead to lamellar bone formation in a rabbit model, while tissue fibrosis was inhibited [374]. A similar study with a silicon dioxide nanoparticle-loaded, non-resorbable membrane that was either functionalized with zinc or doxycycline revealed induction of osteogenic marker genes in cell culture experiments, while simultaneously suppressing RANK-L expression [375,376]. Clinical data from a 5-year period of patients treated with GBR materials also revealed promising outcomes [377].

New generations of GBR materials even offer in situ mineralization and thus stiffening of membranes, which is important in the context of cellular mechanosensing. According to Li et al., the modification of a Bio-Guide^®^ membrane with polyacrylic acid led to self-mineralization of the material after transplantation in a murine bone defect model. Additionally, osteogenesis from mesenchymal stem cells was induced. Molecularly, a clear nuclear accumulation of both YAP and TAZ was registered, which shows both the decisive role of both proteins for osteogenesis and the applicability of such dynamic materials for regenerative purposes [378]. The complex histological composition of the periodontium will, therefore, be an optimal candidate to apply such self-regulating biomaterials, which is especially important regarding tissue–tissue interfaces [148,379].

One major challenge in the application of GBR remains the fact that cells behave differently on 2D or 3D substrates, as already mentioned in the beginning of this section. As above, the actual “cell-guidance”, i.e., the induction of the desired cell behavior, is strongly connected to the material’s properties and topography. Therefore, biophysical and biochemical cues of biomaterials need to be carefully selected in order to enable the clinical success of periodontal regeneration via GBR and other methods. Matrix stiffness, pore size and porosity, the nanotopography, and the stress–relaxation behavior need to be considered from a biomechanical point of view. From the biochemical perspective, the incorporation of growth factors, cytokines, small bioactive molecules, ions, genetic information or other compounds relevant to cellular physiology is noteworthy [380,381,382,383].

In 2D systems, different materials have been tested for their capacity to guide cell behavior. Generally, guided adhesion, migration, spreading, proliferation, differentiation, and stemness maintenance can be distinguished. Adhesion is established via material composition and stiffness. Migration results from compositional gradients, which induce chemotaxis, or mechanical stimuli, which induce mechanotaxis. Cell spreading is the consequence of positive charges or a moderate material hydrophobicity. Compositional motifs are able to stimulate proliferation, while a tissue-like stiffness induces differentiation. In general, soft substrates support stemness maintenance [384]. For instance, graphene-based materials induce osteogenic differentiation of hMSCs [385]. As above, they sustain cell adhesion, spreading, and proliferation. Boron nitride, Tungsten disulfide or molybdenum disulfide materials are other examples for such cell-instructive 2D materials [386]. The exact molecular interplay with the herein presented MT pathways, however, remains to be elucidated.

Studies on 3D-directed cell migration on biocompatible polymers offer great potential to further study cell-guidance. Compared to 2D materials, the third dimensions offer the possibility to construct a certain nanotopography, which feeds back on cellular physiology. For instance, photopolymers are used in fabrication techniques known as laser ablation or direct laser writing (multiphoton lithography). This enables researchers to generate a broad variety of biomaterials with complex 3D nanotopographies. Other possibilities for scaffold preparation include freeze-drying, electrospinning, or 3D printing [387]. Consequently, cell behavior changes upon contact with the material in response to the geometry and physical properties. Exemplarily, Cheng and colleagues observed cell migration parallel to ridges, also known as contact guidance, on a material fabricated with direct laser writing. Therefore, this technique offers the possibility for 3D spatial control of cell behavior [388]. Controlled cell-alignment, which is important for tissue organization, especially in complex composite organs such as the periodontium, is also achievable on such substrates [387]. By using materials with varying matrix stiffnesses, an effect called durotaxis can be observed, meaning that cells migrate from softer to stiffer regions. The incorporation of such a “strain field” in combination with the material’s nanotopography enables even more control on cell behavior and guidance [389]. The exact molecular signaling events that occur during these processes are so far only incompletely understood. Of interest, laser-guided direct writing is not limited to the fabrication process of the biomaterial, but optical forces can be directly used to deposit cells. Thus, cell-to-cell distances and the spatial control of cell-to-cell interaction as well as the interplay of different cell types can be experimentally manipulated [390]. Thus, GBR in combination with the above-discussed properties of 2D and 3D materials in the context of cell guidance in the periodontium harbor great potential to improve oral regenerative therapy approaches.

NMT is less well understood in the context of periodontal health, pathology, and regeneration. Further molecular studies on the regulation of nuclear mechanics and its interplay with other mechanobiological players will shed light into its mechanisms of regulation. In turn, the elucidation of such interplay-governing mechanisms and their underlying molecules opens the road for the discovery of potential targets for therapeutic approaches. As Lamins and Emerin are subjected to phosphorylation, it is tempting to speculate that pharmacologic inhibition of kinases, for instance with currently available tyrosine kinase inhibitors (TKI), will influence NMT and related signaling processes [391]. Screening for mechanobiological consequences of TKI application could clarify regulatory principles of NMT. This is also interesting regarding the many TKIs that are currently applied in cancer therapy [392,393].

To summarize, the discussed principles of therapeutically addressing MT in the context of periodontal homeostasis and regeneration have again demonstrated the importance and complexity of the interconnectedness of MT and NMT, thereby rendering it a fascinating issue of basic and translational research to achieve and/or improve oral tissue regeneration. Apart from growth factors, endocrine stimuli, and the general extracellular milieu, it is of great importance to consider the various mechanisms of actions of MT-inherent mechanobiological signaling and its related processes. In the future, MT will be an indispensable cornerstone of research with respect to oral regenerative medicine.

## 7. Conclusions

MT-immanent mechanobiological signals are key determinants of cell behavior and tissue adaptation to the external cell and tissue environment. The interplay of molecular mechanosensors and mechanotransducers is complex and represents the tight interrelationship between different cellular compartments and signaling hubs. FAs, AJs, YAP/TAZ, the cytoskeleton, LINC, and the nucleoskeleton only represent a small subset of cellular players involved in MT and NMT but reveal important principles of how the cell percepts and integrates biophysical mechanical information. Periodontal tissues are of special interest to MT research and are a paradigm of how the above-discussed signaling networks, cascades, and molecules govern tissue development, homeostasis and regeneration, cell proliferation, differentiation, and pathologic processes, such as periodontitis or OSCC. By further elucidating the cell-type specific and spatiotemporal fine-tuning of mechanobiological processes, the future translation of these principles into clinical applications will prove to be a strong tool in the field of oral regenerative medicine.

## Figures and Tables

**Table 1:** Selected integrin heterodimers and their corresponding ligands.

Integrin Heterodimers	α1β1	α2β1	α3β1	α5β1	αVβ1	αVβ3	αVβ5
Ligand(s)	Different collagens (e.g. type I)	Different collagens (e.g. type I)	laminins	fibronectin	fibronectin	fibronectin	vitronectin
